# An approach to the management of chronic myeloid leukemia in British Columbia

**DOI:** 10.3747/co.v15i2.224

**Published:** 2008-04

**Authors:** D.L. Forrest, X. Jiang, C.J. Eaves, C.L. Smith

**Affiliations:** * Leukemia/BMT Program of BC, Division of Hematology, General Hospital, British Columbia Cancer Agency, and University of British Columbia, Vancouver, BC; † Terry Fox Laboratory, British Columbia Cancer Agency, and Department of Medical Genetics, University of British Columbia, Vancouver, BC

**Keywords:** Chronic myeloid leukemia, treatment guidelines

## Abstract

Chronic myeloid leukemia (cml) is a myeloproliferative disorder whose therapy has changed dramatically since the late 1990s. With the introduction of the tyrosine kinase inhibitor (tki) imatinib mesylate, the treatment outcomes for patients with cml have improved markedly, and hematopoietic stem-cell transplantation is no longer routinely offered as first-line therapy for most patients in chronic phase.

However, resistance to tki therapy is increasingly being recognized, and alternative therapy is needed for this group of patients. In addition, the development of models predicting response to tki therapy is desired, so that appropriate treatment strategies can be used for individual patients. The present report serves to outline the approach to the treatment of cml in British Columbia and to highlight areas of ongoing research.

## 1. INTRODUCTION

Chronic myeloid leukemia (cml) is a myeloproliferative disorder characterized by the increased proliferation of myeloid cells at all stages of maturation. The cytogenetic hallmark of the disease is the presence of the Philadelphia (Ph) chromosome or its molecular equivalent (*BCR-ABL*). The Ph chromosome consists of a reciprocal translocation between the Ableson oncogene (*ABL*) on chromosome 9 and the breakpoint cluster region (*BCR*) on chromosome 22, resulting in a chimeric Bcr-Abl protein with constitutive tyrosine kinase activity. The protein kinase is instrumental in the activation of multiple signalling pathways (JAK– STAT, RAS, and PI3K) affecting cellular proliferation and apoptosis, leading to a leukemic phenotype.

With the greater understanding of the molecular biology of cml has come the development of novel therapies specifically designed to selectively inhibit the tyrosine kinase activity of Bcr-Abl. Indeed, with the introduction of such targeted therapy, the therapeutic options and management strategies for cml have changed considerably in the last few years. Allogeneic stem-cell transplantation remains a potentially curative treatment for selected patients with cml, but the lack of suitable donors and the associated morbidity and mortality limits its applicability. The use of tyrosine kinase inhibitors (tkis) in cml has so far demonstrated impressive results with minimal toxicity and has thus drastically changed the landscape of cml therapy. However, the lack of long-term clinical data— in particular, data relating to survival—has led to an ongoing controversy about the best therapy for individual patients.

The aims of the present report are to outline the approach to the management of cml in British Columbia and to discuss the challenges that remain for future research.

## 2. DISCUSSION

### 2.1 Diagnosis and Initial Investigations

At presentation, initial investigations should include a complete blood count and white blood cell differential; serum electrolytes, including creatinine; liver function tests; uric acid; and a bone marrow aspirate and biopsy with cytogenetic analysis. Bone marrow examination provides valuable information regarding cellular morphology, marrow cellularity, number of blast cells, and the presence of fibrosis. Standard karyotyping is useful to document the presence of the Ph chromosome and whether additional chromosomal abnormalities are present at diagnosis, constituting clonal evolution. Taken together, this information will allow the patient to be appropriately classified into chronic phase (cp), accelerated phase (ap), or blast phase (bp), and to be assigned a Sokal risk score 1.

Further studies that may be useful include fluorescent *in situ* hybridization (fish), which will detect the presence or absence of deletions in the derivative 9 chromosome. Such deletions have been associated with inferior overall survival and progression-free survival for cp patients treated with interferon alpha 2. Less is known about the impact of such deletions for patients receiving imatinib therapy, although preliminary reports suggest little to no effect on outcome 3, 4.

Molecular analysis of the *BCR-ABL* fusion gene by polymerase chain reaction (pcr) can also provide confirmation of the presence of the Ph chromosome, pcr being associated with a high degree of sensitivity and specificity. At diagnosis, pcr positivity for *BCRABL* transcripts on blood or bone marrow can confirm the diagnosis of cml even in the rare patient that is Ph-negative by standard karyotyping or fish analysis. Furthermore, quantitation by pcr of the *BCR-ABL* transcripts (qpcr) can provide a baseline measurement for subsequent disease monitoring. The high degree of sensitivity of qpcr allows for monitoring of minimal residual disease for patients achieving a complete cytogenetic response (ccr) to therapy. Because most cp patients achieve a ccr on imatinib, qpcr is indispensable as a monitoring strategy in cml. By virtue of its ability to detect small changes in the disease burden, it may reveal early signs of emerging resistance to therapy before a hematologic or cytogenetic relapse occurs.

### 2.2 Prognostic Factors

In the pre-imatinib era, several clinical and laboratory features were identified that predict response to therapy. The Sokal score, initially developed to predict response to busulfan still retains some predictive value with imatinib therapy 1. Patients with a high-risk Sokal score have a 69% chance of achieving ccr, as compared with an 82% chance with an intermediate score or a 98% chance with a low-risk score 5. However, once a ccr has been achieved, the Sokal score loses its prognostic importance, and progression-free survival for all three groups remains equivalent. Therefore, even a high-risk Sokal score at diagnosis does not on its own seem to justify an alternative front-line therapy without an initial trial of imatinib.

The presence of karyotypic abnormalities at diagnosis in addition to the standard Ph chromosome has also been associated with an adverse prognosis. Clonal evolution is usually accompanied by other manifestations of more advanced-phase disease; however, in isolation, clonal evolution may not necessarily be associated with an inferior outcome on higher-dose imatinib 6. Furthermore, deletions involving the derivative 9 chromosome determined by fish analysis are also predictive of poor outcome with interferon-based therapy. However, the significance of such deletions is less clear for patients receiving imatinib, and preliminary reports, at least, suggest little to no effect on outcome ^3, 4.^

These historical clinical and laboratory factors have limited predictive value for patients now receiving therapy with imatinib, and “newer” predictive models are needed. Because much of the biology of cml, including resistance to tkis, can be explained by the biology of the cml stem cells that sustain the cml clone, examining various aspects of the stem-cell compartment will hopefully help to determine if any stem-cell-related factors are predictive of patterns of failure to tki therapy (see “Future Strategies,” later in this article).

### 2.3 Therapeutic Options and Treatment Algorithms

**FIGURE 1:**
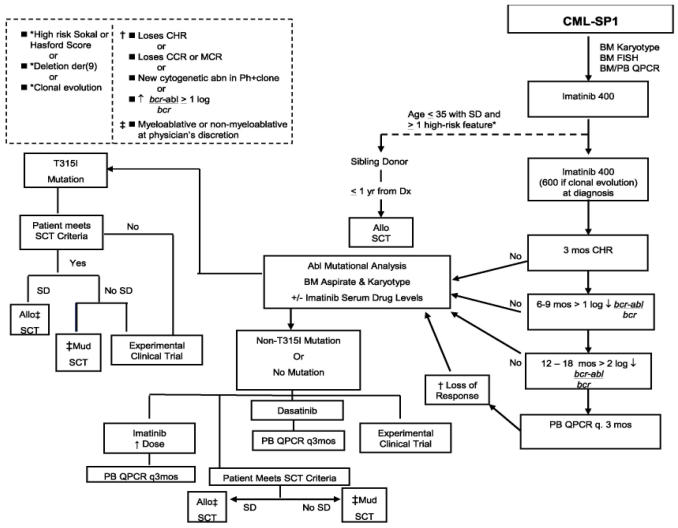
Treatment algorithm for chronic myeloid leukemia (cml) in chronic (stable) phase (sp). chr = complete hematologic response; mcr = major cytogenetic response; Ph+ = positive for the Philadelphia chromosome; bm = bone marrow; fish = fluorescence *in situ* hybridization; pb = peripheral blood; qpcr = quantitative polymerase chain reaction; sd = sibling donor; Dx = diagnosis; q. = every; Allo sct = allogeneic stemcell transplantation; Mud sct = matched unrelated donor stem-cell transplantation.

**FIGURE 2:**
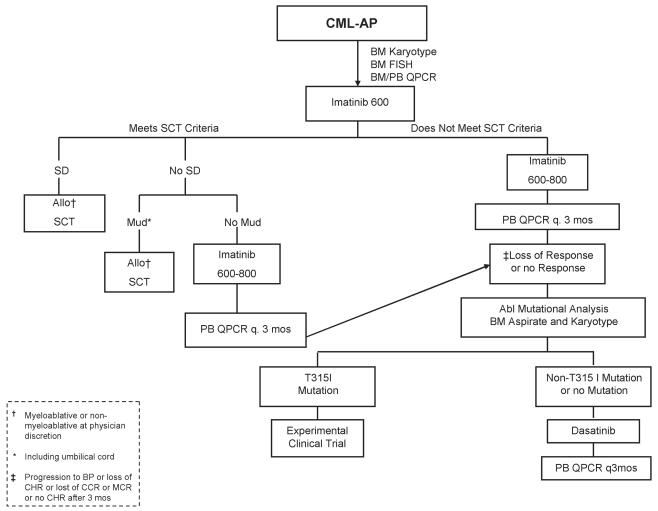
Treatment algorithm for chronic myeloid leukemia (cml) in accelerated phase (ap). bm = bone marrow; fish = fluorescence *in situ* hybridization; pb = peripheral blood; qpcr = quantitative polymerase chain reaction; sct = stem-cell transplantation; sd = sibling donor; Mud = matched unrelated donor; Allo = allogeneic; bp = blast phase; chr = complete hematologic response; mcr = major cytogenetic response.

**FIGURE 3:**
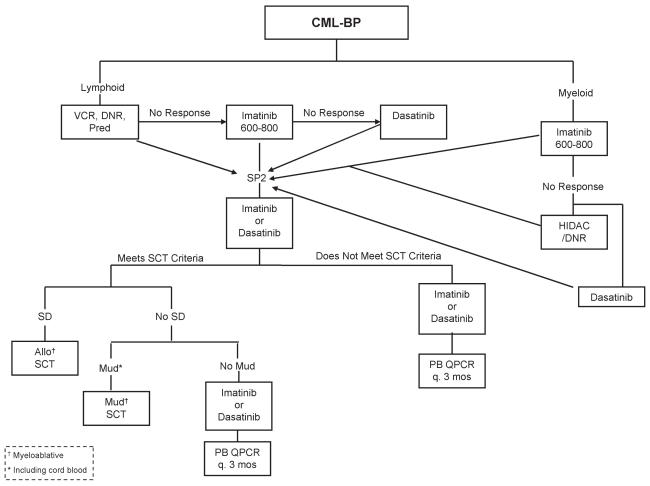
Treatment algorithm for chronic myeloid leukemia (cml) in blast phase (bp). vcr = vincristine; dnr = daunorubicin; Pred = prednisone; sp2 = second stable phase; hidac = high-dose cytarabine; sd = sibling donor; Allo = allogeneic; sct = stem-cell transplantation; Mud = matched unrelated donor; pb = peripheral blood; qpcr = quantitative polymerase chain reaction.

With the introduction of tkis (imatinib, dasatinib, nilotinib), much has changed in the standard approach to cml therapy. Before imatinib became available, most cml patients received treatment with interferon alpha, with or without cytarabine, or alternatively underwent allogeneic stem-cell transplantation if deemed eligible with a suitable donor. As compared with hydroxyurea or busulfan, interferon alpha has been shown to prolong survival; however, only a small proportion of patients (20%–30%) achieve a major cytogenetic response, and the survival benefit is largely limited to such responders 7, 8. Furthermore, interferon is associated with a number of dose-related toxicities, and most patients require dose reductions or discontinuation. For those reasons, interferon therapy has been largely replaced by the more effective and less toxic tkis. In British Columbia, we have developed treatment algorithms (Figures 1, 2, and 3) that are largely based on the newly developed tki therapy.

### 2.4 Imatinib Mesylate

Imatinib was the first tki approved in Canada for the treatment of cml in all phases of disease. The pivotal clinical trial establishing efficacy for imatinib was the International Randomized Study of Interferon and STI571, an open-label phase iii randomized study of 1106 patients with newly diagnosed cml in cp 5. After a median follow-up of 60 months, survival without progression to ap or bp was 93% for imatinib, and the estimated rate of ccr was 87%. By contrast, only 3% of patients initially randomized to interferon and cytarabine continued on their initial therapy.

Given those excellent results, our approach in British Columbia is to offer imatinib at the recommended starting dose of 400 mg daily as initial therapy to all patients newly diagnosed with cml in cp. Patients with cytogenetic abnormalities in addition to the Ph chromosome at diagnosis (clonal evolution), but with no other features of ap, are treated with escalated-dose imatinib (600 mg daily). The rational for escalation is that, although clonal evolution is usually accompanied by other features of more advanced disease, clonal evolution by itself may not necessarily be associated with an inferior outcome on higher-dose imatinib 6.

The subsequent management of patients on imatinib is guided by their response to therapy as determined by hematologic, cytogenetic, and molecular measurements over time (see “Disease Monitoring and Imatinib Resistance” later in this article).

### 2.5 Hematopoietic Stem-Cell Transplantation

**FIGURE 4:**
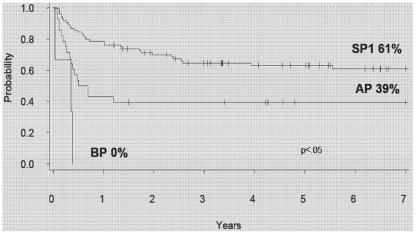
Event-free survival for related-donor stem-cell transplantation in chronic myeloid leukemia (cml) by disease status at 7 years. sp1 = first stable phase; ap = accelerated phase; bp = blast phase.

Allogeneic hematopoietic stem-cell transplantation (hsct) remains the only known potentially curative therapy for cml, and before the introduction of imatinib, hsct was routinely offered to eligible patients with suitable donors. Data from the Leukemia/BMT Program of BC shows that the 7-year event-free survival for related donor allogeneic transplant in cp is 61% (Figure 4) with a 7-year transplant-related mortality of 29% and a risk of relapse of 14%. Since the late 1990s, improvements in supportive care have resulted in lower transplant-related mortality and improved outcomes; however, the remaining risks of the procedure and a lack of suitable donors has in many cases limited its applicability—at least as initial therapy for newly diagnosed patients. With availability of tkis, the potential toxicity of a transplant must now be balanced

With the chance of long-term survival. With that balance in mind, attempts have been made to better assess the risks and benefits for patients eligible for hsct by developing scoring systems aimed at predicting transplant outcome for selected patients 9. However, despite an ability to identify patients predicted to have a low risk of transplant-related mortality, an initial trial of imatinib therapy is still justified for nearly all patients; hsct is reserved for patients without a satisfactory response to imatinib therapy. That being said, the final decision concerning whether to pursue medical management or transplantation should be discussed with each patient at diagnosis so that therapy can be appropriately individualized. In British Columbia, referral to the Leukemia/BMT Program of BC of every patient considered a potential transplant candidate for a detailed discussion about the role of transplantation in their management is strongly encouraged.

### 2.6 Disease Monitoring and Imatinib Resistance

Once patients have commenced imatinib therapy, our approach in British Columbia is to document normalization of peripheral blood counts [complete hematologic response (chr)], which ideally should occur within 3 months of the start of imatinib therapy. Once chr has been obtained, further evaluation of disease response requires cytogenetic or fish analysis on bone marrow samples or, alternatively, qpcr of *BCR-ABL* on blood or marrow.

Cytogenetic analysis is labour-intensive, requires serial bone marrow aspirates, and has limited sensitivity; therefore, in British Columbia, we have elected to use qpcr to monitor all of our cml patients every 3 months once a chr has been obtained. This highly sensitive test is able to detect 1 abnormal Ph+ cell in 10,000 normal cells, and it has the further advantage of being able to be performed on peripheral blood. The correlation of peripheral blood qpcr with bone marrow cytogenetics is excellent and thus provides a simple, but very sensitive tool to measure minimal residual disease 10, 11. In British Columbia, routine annual bone marrow tests are no longer recommended, provided that response to therapy is satisfactory (chr by 3 months, >1-log reduction in *BCR-ABL* by 6–12 months, >2-log reduction in *BCR-ABL* by 12–18 months; Table I) 12.

For patients who do not achieve those milestones or who later develop progression (resistance) after an initial response, a change in therapy is needed. It is important to note that there is no evidence that tki therapy actually “causes” resistant mutant *BCR-ABL* cells to arise; rather, the therapy appears to select for mutants already present. Once resistance (either primary or acquired) to imatinib has been documented (Table II), we recommend a bone marrow aspirate and biopsy with cytogenetic analysis to establish current disease status and whether evidence of progression to ap or bp is present. Mutational analysis should also be undertaken to document the presence or absence of mutations in the *BCR-ABL* kinase domain that may mediate imatinib resistance 13. Finally, a trough serum imatinib level may also provide useful information 14. Patients with low serum imatinib levels and inferior responses may respond to escalated-dose imatinib.

Depending on the results of the foregoing investigations, a change in therapy may be undertaken, which may include dose escalation of imatinib, a change to dasatinib, allogeneic hsct, or enrolment in a clinical trial.

### 2.7 Dasatinib

Dasatinib is a dual tki and Src inhibitor with increased potency as compared with imatinib. It recently received conditional approval from Health Canada for the treatment of cml (cp, ap, or bp) in patients with resistance or intolerance to imatinib therapy.

Primary resistance (lack of efficacy from the onset of therapy) to imatinib is unusual in cp patients, but acquired resistance resulting from the selection and expansion of pre-existing mutants can be seen in approximately 15% of cp patients. Such resistance increases in frequency with more advanced phases of disease. The most common mechanism of acquired resistance to imatinib is point mutations in the *ABL* kinase domain, interfering with imatinib binding; however, *BCR-ABL* genomic amplification and alteration in drug efflux and influx have also been implicated 13. The recent phase ii studies of dasatinib therapy for imatinib-resistant or -intolerant cml patients documented efficacy in all phases of disease, with an acceptable side-effect profile 15–17. Based on the results of those clinical trials, the British Columbia Cancer Agency recently approved the use of dasatinib for cml patients (cp, ap, and bp) with documented resistance or intolerance to imatinib therapy (Table II)

One special group of patients, those harbouring a T315I mutation in the *ABL* kinase domain, warrants further discussion. The T315I mutation has been shown to mediate resistance to all three tkis (imatinib, dasatinib, nilotinib), and therefore patients with this particular mutation should be evaluated for an allogeneic hsct or offered experimental therapy if transplantation is not a suitable option.

### 2.8 Nilotinib

Like dasatinib, nilotinib is a new, second-generation tki with structural similarity to imatinib. It has greater potency than imatinib, with activity against most imatinibresistant *BCR-ABL* mutations, with the notable exception of the T315I mutation, in which resistance to tki therapy is universal. Results of phase i and ii studies with nilotinib are encouraging, with a chr of 75% and a ccr of 40% in cp patients with imatinib resistance or intolerance 18, 19. However, longer follow-up will be needed to assess whether these responses are durable. Based on the early results, further clinical trials with nilotinib are underway, but nilotinib is not currently approved by Health Canada. Access therefore remains in the context of clinical trials.

## 3. FUTURE STRATEGIES

Despite the excellent clinical activity of the tkis (imatinib, dasatinib, nilotinib), these drugs are generally ineffective on the cml stem-cell population, which includes a significant quiescent component. That particular cell population is also felt to represent the reservoir from which clinical resistance may develop. As a consequence of the persistence of cml stem cells, tki therapy is not considered curative, and new treatment strategies continue to be explored.

One strategy currently undergoing exploration is the targeting of molecular pathways activated by the Bcr-Abl oncoprotein in the cml stem cells. The farnesyl transferase inhibitors (ftis) are a new class of anticancer drugs in this area. These drugs competitively inhibit the enzyme farnesyl transferase. The potent cytotoxic fti BMS-214662 (Bristol–Myers Squibb, Montreal, QC) has antitumour activity in several tumour models. Recent work by a group at the University of Glasgow has also shown that *in vitro* treatment with BMS-214662 results in a significant reduction in the number of cml stem cells, suggesting a possible new avenue to treatment.

Approximately 15% of cp patients receiving imatinib will develop resistance, and the frequency increases (to as high as 40%) in more advanced phases. Identification at diagnosis, through new predictive tests, of patients that are likely to develop tki resistance so that an alternative therapy may be contemplated is another important need. To that end, we are investigating whether cml stem cells from imatinib non-responders have an increased inherent imatinib insensitivity or genetic instability that predisposes to the acquisition of *BCR-ABL* mutations 20. Our preliminary results suggest that analysis of specific properties of the cml stem cells at diagnosis may help to predict individual patient response to tki therapy and thus aid in the future development of more effective treatment algorithms.

All of this research work depends on the provision of appropriate clinical material to the laboratory, and as part of our treatment strategy in British Columbia, all patients with cml are asked to participate in these efforts by providing blood and marrow samples at diagnosis once consent has been obtained. Cooperation of this kind between the patient, the clinician, and the laboratory will help in the ultimate discovery of more effective treatments for all patients diagnosed with cml.

## Figures and Tables

**TABLE I:** Milestone criteria and timelines

Response	Duration of therapy
Complete hematologic response	3 months
Major cytogenetic response	6–12 months
Complete cytogenetic response	12–18 months
Major molecular response	18–24 months

**TABLE II:** Resistance and intolerance to imatinib

RESISTANCE	
cml chronic phase	cml accelerated and blast phases
Lack of complete hematologic response after 3 months a Lack of any cytogenetic response after 6 months a Lack of major cytogenetic response (or 1-log reduction *BCR-ABL*) after 12 months a Lack of complete cytogenetic response (or 2-log reduction *BCR-ABL*) after 18 months a Cytogenetic relapse (loss of complete cytogenetic response or >2-log reduction of *BCR-ABL,* or major cytogenetic response or >1-log reduction of *BCR-ABL,* or any Ph+ cell increase ≥ 30%) Loss of complete hematologic response Progression to accelerated or blast phase CML for ≥ 4 weeks, unless intolerant	Lack of response after ≥ 4 weeks of imatinib ≥ 600 mg No complete hematologic response in accelerated phase at 3 months Incomplete response with no further improvement in blast phase after 1 month of imatinib ≥ 600 mg daily Cytogenetic relapse (loss of complete cytogenetic response or >2-log reduction of *BCR-ABL,* or major cytogenetic response or >1-log reduction of *BCR-ABL,* or any Ph+ cell increase ≥ 30%) Loss of complete hematologic response Progression of accelerated phase to blast phase or to recurrent blast phase

INTOLERANCE	

≥ Grade 3 non-hematologic toxicity not responding to symptomatic treatment or dose adjustments of imatinib
Grade 4 hematologic toxicity lasting >7 days
Sustained highly symptomatic grade 2 non-hematologic toxicity

a Patient should be treated with a minimum of 600 mg imatinib.

cml = chronic myeloid leukemia; Ph+ = positive for the Philadelphia chromosome.
